# Prehabilitation with wearables versus standard of care before major abdominal cancer surgery: a randomised controlled pilot study (trial registration: NCT04047524)

**DOI:** 10.1007/s00464-021-08365-6

**Published:** 2021-03-15

**Authors:** Ellen Waller, Paul Sutton, Seema Rahman, Jonathan Allen, John Saxton, Omer Aziz

**Affiliations:** 1 Faculty of Biology, Medicine and Health, The University of Manchester, Manchester, UK; 2 Colorectal and Peritoneal Oncology Centre, The Christie NHS Foundation Trust, Manchester, M20 4BX UK; 3 Department of Sport, Exercise, and Rehabilitation, Northumbria University, Newcastle Upon Tyne, UK

**Keywords:** Exercise, Fitness trackers, Prehabilitation, Pre-operative, Smartwatches, Wearables

## Abstract

**Background:**

Prehabilitation aims to improve post-operative outcomes by enhancing pre-operative fitness but is labour-intensive. This pilot study aimed to assess the efficacy of a tri-modal prehabilitation programme delivered by smartwatches for improving functional fitness prior to major abdominal cancer surgery.

**Methods:**

A single-centre, randomised controlled pilot study, in which 22 patients were randomised to: (a) a prehabilitation group (*n* = 11), comprising of home-based exercise, nutritional, and dietary advice delivered using a wrist-worn smartwatch connected to a smartphone application; or (b) a control group (*n* = 11) receiving usual care, with patients given a smartwatch as a placebo. Eligible participants had over two weeks until planned surgery. The primary outcome was pre-operative physical activity including 6-min walk test (6MWT) distance, with secondary outcomes including change in body weight and hospital anxiety and depression score (HADS).

**Results:**

Recruitment was 67% of eligible patients, with groups matched for baseline characteristics. The prehabilitation group engaged in more daily minutes of moderate [25.1 min (95% CI 9.79–40.44) vs 13.1 min (95% CI 5.97–20.31), *p* = 0.063] and vigorous physical activity [36.1 min (95% CI 21.24–50.90) vs 17.5 min (95% CI 5.18–29.73), *p* = 0.022] compared to controls. They also had significantly greater improvements in 6MWT distance compared to controls [+ 85.6 m (95% CI, + 18.06 to + 153.21) vs + 13.23 m (95% CI − 6.78 to 33.23), *p* = 0.014]. HADS scores remained unchanged from baseline in both groups.

**Conclusion:**

Prehabilitation in the colorectal cancer care setting can be delivered using smartwatches and mobile applications. Furthermore, this study provides early indicative evidence that such technologies can improve functional capacity prior to surgery

**Trial registration:**

NCT04047524.

**Supplementary Information:**

The online version contains supplementary material available at 10.1007/s00464-021-08365-6.

In the UK, patients undergoing major colorectal cancer surgery have 3% 90-day mortality and an 11% unplanned readmission rate from post-operative complications [1]. Tri-modal prehabilitation aims to enhance post-operative recovery by improving patients’ pre-operative functional capacity, nutritional status and psychological readiness for surgery via exercise, dietary, and psychological support [2]. Delivery of prehabilitation before major abdominal cancer surgery has ranged from unsupervised home-based interventions (exercise booklets, and CDs) and computer programs, to supervised hospital-based programmes using cycle ergometers and exercise equipment [3, 4]. Unsupervised home-based programmes are less labour-intensive and more cost-effective but are reported to have lower compliance rates (16–87%) [4, 5].

The impact of prehabilitation on functional capacity can be assessed using the 6-min walk test (6MWT) [6]. A clinically meaningful increase in 6MWT distance is reported to be 20 m [7–9] following 3–6 weeks of home-based prehabilitation [10, 11]. A recent pooled analysis of comparative studies suggests this may result a 5-year disease free survival in stage III colorectal cancer patients from 50.9% without prehabilitation versus 73.4% with (*p* = 0.044) [12]. Delivery of prehabilitation across a healthcare system however, remains a challenge [13]. Wearable technologies linked to smartphones through software applications can promote health behaviour change including physical activity, rehabilitation, weight loss, and may be ideally suited to delivering prehabilitation [14–16]. This prospective randomised controlled pilot study aimed to determine whether tri-modal prehabilitation delivered via a wearable technology can successfully increase pre-operative physical activity levels and improve pre-operative functional capacity in patients undergoing major abdominal cancer surgery.

## Materials and methods

This was a single-centre, parallel-arm randomised controlled pilot study (NCT04047524). During the study period consecutive patients undergoing major abdominal cancer surgery at a tertiary cancer centre (The Christie NHS Foundation Trust, Manchester, UK) were randomised (1:1) to the intervention or control group. A sample size of 15 in each group was calculated based on the ability to detect an increase in the primary outcome 6MWT distance of 20 m in the prehabilitation versus the standard care group. The intervention group received a prehabilitation programme delivered using a Fitbit Smartwatch (Fitbit Inc, San Francisco, CA, USA) with a digital display and Smartphone Application (App). The control group received a Fitbit smartwatch with no display or patient feedback as a placebo. This meant comparable activity data was collected in both groups. Clinical aspects of care were not altered for either group. The study was approved by Health and Social Care REC A on 25/02/2019 and Health Research Authority on 05/03/2019. Written informed consent was obtained from all participants prior to enrolment.

### Inclusion criteria

Participants had to be aged 18 years or over, able to consent to participate in the prehabilitation programme, be undergoing major abdominal cancer surgery, have at least 2 weeks to their operation date, and be able to understand written and spoken English.

### Exclusion criteria

Participants were excluded if they had any health conditions which prevented them from safely taking part in a home-based exercise programme. These included patients who had within the past 3 months had a myocardial infarction or stroke. Participants were also excluded if they were already active users of an activity monitoring smartwatch. Patients were not excluded if they were already completing the UK national recommendation of 150 min physical activity per week [17] to avoid selection bias.

### Randomisation

A random sequence with 15 participants in each study group was generated using the online randomisation software “GraphPad” (GraphPad Software Inc, San Diego, CA, USA). This sequence was placed into sequentially numbered sealed opaque envelopes. After participants gave their fully informed consent and completed baseline questionnaires and observations, the appropriate sequential envelope was opened to obtain group allocation. All participants then completed baseline 6MWT.

### Blinding

All patients approached were informed that the study was investigating whether prehabilitation could have a role to play in improving pre-operative fitness. It was clearly explained that allocation to the control or prehabilitation arm of the study would not knowingly advantage or disadvantage them in any way. Whilst both groups were given wearable devices (placebo in the control group as outlined below), the inherent differences between the intervention and control groups made blinding challenging for study participants. The baseline 6MWT distance was used by the physiotherapist to determine baseline fitness in the prehabilitation group participants in order to help tailor the intensity of their physical activity.

### Wearable activity monitors

A Fitbit Charge 2 smartwatch was provided to each participant in the prehabilitation group. This has a screen that allows the wearer to self-monitor real-time physical activity levels. A Fitbit Flex 2 (placebo device) was provided to each participant in the control group. This is a band without a screen that can be set to not provide feedback on physical activity levels to the wearer. Both smartwatches record daily steps and duration and intensity of physical activity, which can be viewed via the Fitbit App (https://www.fitbit.com), which was downloaded onto participants’ smartphones (participants without a smartphone were loaned one). All participants were instructed to wear their device all days of the pre-operative period and received instructions on how to upload smartwatch data to the Fitbit App daily using Bluetooth wireless technology. In the control group the App was only used to collect data from their Fitbit with no feedback given. Data from the control group were used as an objective measure of daily physical activity levels in a sample of patients not provided with a prehabilitation programme, for comparison with the same data from the intervention group.

### Tri-modal prehabilitation group intervention

Prehabilitation group participants were shown how to use the Fitbit Charge 2 device and Fitbit App as a motivational tool for increasing daily physical activity levels and were shown how to use the Fitbit App food log to support dietary behaviour change. A separate mindfulness app was provided for stress management. Standardised structured weekly phone calls were provided to allow reporting of technical issues and provide tailored prehabilitation support.

#### Structured exercise and physical activity

**Fig. 1:**
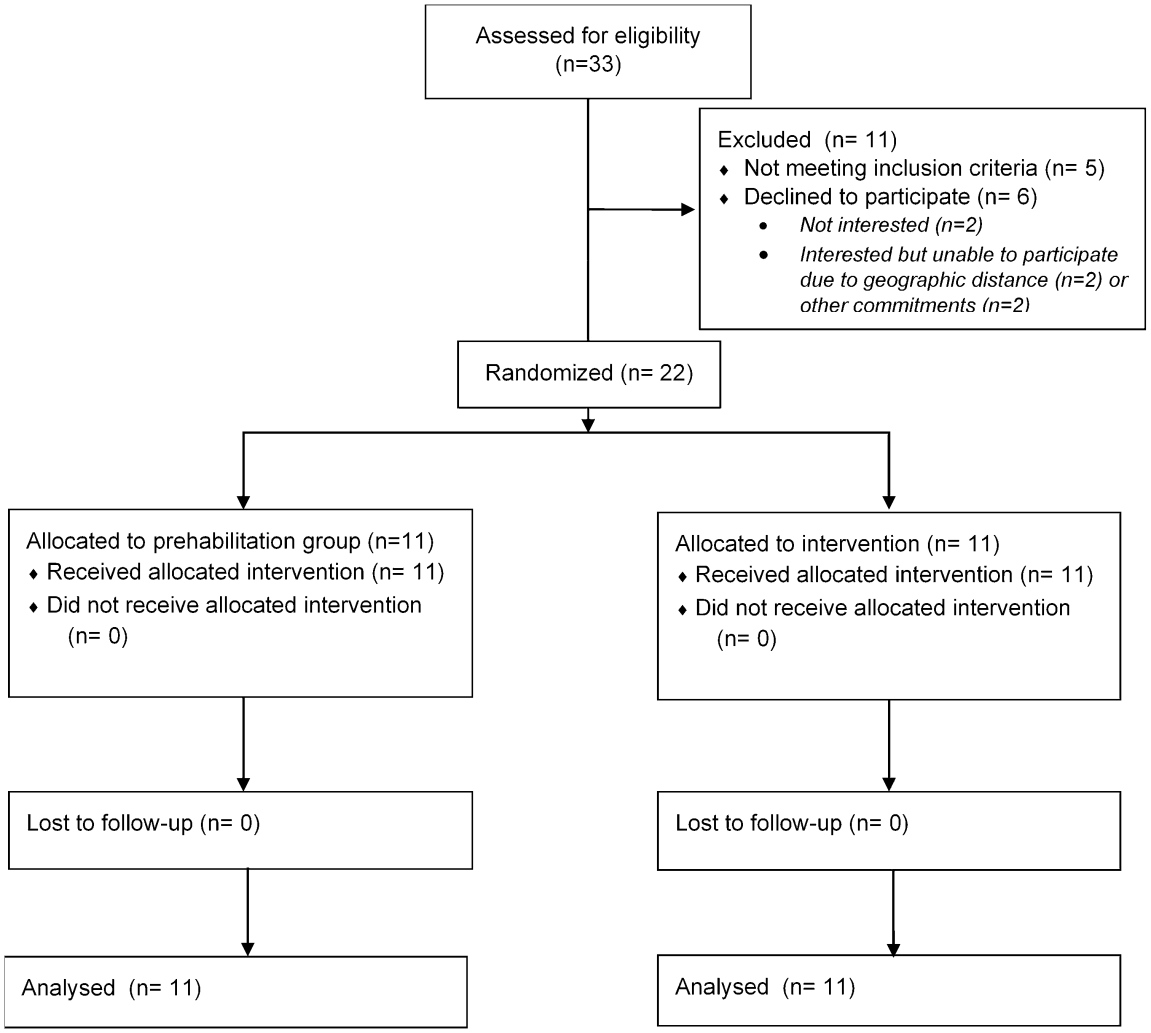
The intensity of exercise programmes provided to the participants

Participants in the prehabilitation group were assessed by a physiotherapist and given an individualised structured exercise and physical activity programme. This comprised aerobic exercise (3 × per week), resistance exercise using a resistance band consisting of 8–10 repetitions in two sets (2 × per week), and encouragement to engage in additional physical activity (e.g. increasing daily walking, use of stairs, etc.) or structured exercise of their choice for 30 min on two other days. The intensity of activity was tailored to each participant’s individual fitness level, as shown in Fig. 1, with the physiotherapist also assessing baseline strength and providing a resistance band of appropriate resistance. Each participant’s target heart rate was calculated using the Karvonen formula and participants were instructed to aim for this target heart rate during the prescribed exercise using the heart rate monitor on the Fitbit Charge 2. Participants were considered to be completing moderate-intensity activity when achieving 50–70% of their predicted maximum heart rate (based on 220—age) [18]. Participants were also provided with a BORG Perceived Exertion Scale and instructed to work at a level of perceived exertion between 12 and 16 indicating “somewhat hard” to “hard” physical activity.

#### Nutrition

Increased protein intake through Oral Nutritional Supplementation has been demonstrated to reduce length of stay by 2 days following colorectal surgery [19] and poor pre-operative nutritional status in cancer patients is associated with sarcopenia and myopenia which increase post-operative complications [20, 21]. Therefore, participants in the prehabilitation group were provided with written dietary advice and watched a presentation on pre-operative nutrition which emphasised the importance of avoiding unintentional weight loss and increasing protein consumption to maintain muscle mass prior to surgery. This advice is presented in Online Appendix. They were shown how to use the Fitbit App food log and encouraged to use this to monitor their daily dietary intake and increase protein intake.

#### Psychosocial support

The peri-operative period is associated with increased levels of anxiety and depression in cancer patients [22, 23]. Pre-operative psychological support may relieve these symptoms and improve Quality of Life for patients awaiting cancer surgery and mindfulness apps have previously been shown to reduce symptoms of anxiety and depression in cancer patients [24, 25]. Participants in the prehabilitation group were therefore instructed to complete one guided meditation per day using a mindfulness app which provides stress management and relaxation techniques (Smiling Mind Pty Ltd—https://www.smilingmind.com.au/smiling-mind-app).

### Outcome measures

The primary outcome was quantitative data collected via a Fitbit aimed at determining the number of participants whose physical activity increased and by how much during the study. This included levels of physical activity (light, moderate, and vigorous) and functional walking capacity as measured by the 6MWT which was conducted according to standard operating procedures at baseline and on the day before surgery [6]. Secondary outcomes included: change in body weight, and change in psychological well-being measured using the Hospital Anxiety and Depression (HADS) questionnaire from baseline to the day before surgery, daily step counts, and participant satisfaction [26]. Daily step counts were determined by the smartwatch accelerometers and active minutes were calculated using estimates of metabolic equivalents after 10 continuous minutes of physical activity. Patient recruitment and retention rates and adverse events were also recorded.

Both the Fitbit Flex 2 and Charge 2 use the same 3-axis accelerometer technology to record activity levels and Fitbit devices have previously demonstrated accuracy and inter-device reliability [27, 28]. Physical activity data were synchronised to an online study dashboard “Fitabase” (Fitabase, San Diego, CA, USA) which allowed the research team to perform retrospective analysis. An Information Governance officer thoroughly reviewed the Fitabase privacy notice (Available from: www.fitabase.com/resources/knowledge-base/working-with-the-irb/data-security-privacy/) to ensure compliance to data security and ethical guidelines and had no concerns relating to the security of the de-identified data. Fitabase is under the Privacy shield framework, and they do not collect IP addresses or GPS data.

### Statistical analysis

Data analysis was undertaken using StatsDirect (StatsDirect Ltd, Merseyside, UK). Statistical significance was defined as a *p* value of < 0.05. A Shapiro–Wilk test was used to determine whether data were normally distributed. Normally distributed data were compared using an independent groups *t* test. Non-normally distributed data were compared using a Mann–Whitney *U* test. Within-group analyses were conducted using a paired *T* test. Qualitative data was generated from end-of-study questionnaires.

## Results

**Fig. 2:**
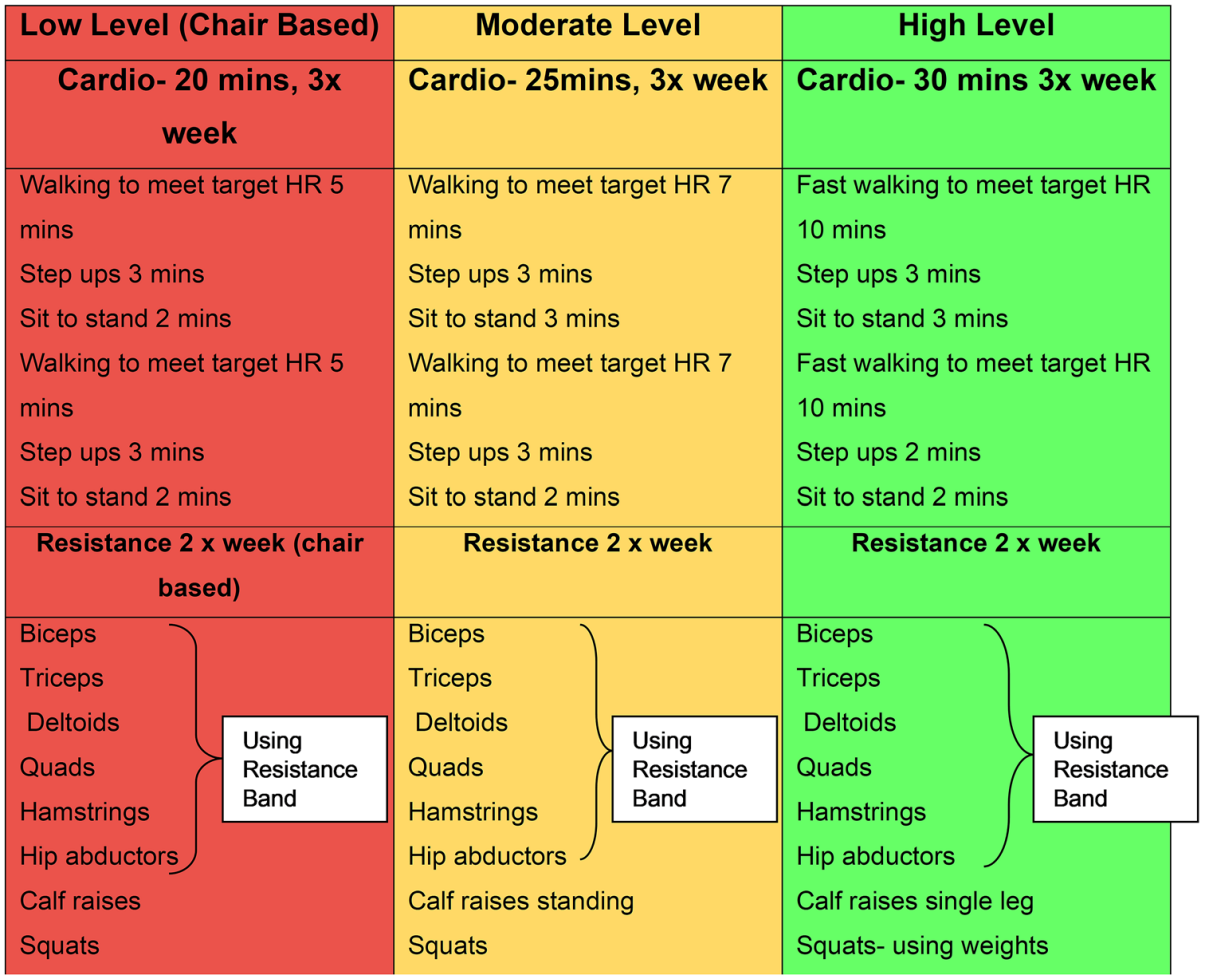
CONSORT flow diagram of participants through the study

This study was open to recruitment between 14/05/2019 and 23/09/2019. A total of 33 participants were identified, of which 11 were either declined or were ineligible, leaving 22 participants randomised during the enrolment period (Fig. 2). The overall recruitment rate was 67% (rising to 79% when excluding patients who did not meet the inclusion criteria). Of note, no patient was excluded because of pre-existing medical conditions. Retention on the study was 100%.

### Baseline characteristics

Baseline characteristics of the prehabilitation and control groups are reported in Table 1. The mean duration from enrolment to the day before surgery was greater in the prehabilitation group at 30.5 (95% CI 18.7–42.2) days versus 20.8 (95% CI, 12.7–29.0) days in the control group (*p* = 0.072), although this was not statistically significant. This difference in days before surgery between the two groups occurred as two prehabilitation group participants had their scheduled surgery date unexpectedly delayed following enrolment. There was no significant difference between the two groups for baseline 6MWT distance or HADS (Table 2).

**Table 1:** Baseline characteristics and surgical details of the prehabilitation and control groups

	Prehabilitation group (*n* = 11)	Control group (*n* = 11)	*p* value
Age (years)	55.5 (49.2, 61.7)	61.0 (53.1, 68.9)	0.223
Age ≥ 65 years	1 (9%)	4 (36%)	
Sex ratio (male:female)	4:7	7:4	
BMI (kg/m^2^)	30.0 (25.6, 34.4)	27.8 (23.4, 32.2)	0.442
ECOG performance status			
0	9 (82%)	10 (91%)	
1	2 (18%)	1 (9%)	
2–4	0 (0%)	0 (0%)	
Primary diagnosis			
Colorectal adenocarcinoma	7 (64%)	8 (73%)	
Pseudomyxoma peritonei	3 (27%)	1 (9%)	
Other	1 (9%)	2 (18%)	
Type of surgery			
CRS and HIPEC	9 (82%)	7 (64%)	
AP resection	0 (0%)	2 (18%)	
Total pelvic clearance	1 (9%)	1 (9%)	
Right hemicolectomy + cystectomy	0 (0%)	1 (9%)	
Laparotomy + small bowel resection	1 (9%)	0 (0%)	
Length of time from study appointment to surgery (days)	30.5 (18.7, 42.2)	20.8 (12.7, 29.0)	0.072

Data are expressed as mean (95% CI), *n* (%), or male:female

*AP* abdominoperineal, *BMI* body mass index, *CRS with HIPEC* cytoreductive surgery and hyperthermic intraperitoneal chemotherapy, *ECOG* Eastern Cooperative Group

**Table 2:** Mean baseline HADS Scores and change in mean 6-min walk test distance from baseline until the day before surgery (pre-operative)

Outcome measure	Prehabilitation group (*n* = 11)	Control group (*n* = 11)	*p* value
Baseline HADS anxiety score	5.8 (3.7, 10.1)	6.6 (3.2, 10.1)	0.658
Baseline HADS depression score	3.1 (1.4, 4.8)	3.7 (1.5, 6.0)	0.622
Baseline 6MWT (metres)	520.9 (450.4, 591.3)	482.6 (433.3, 532.0)	0.156
Pre-operative 6MWT (metres)	606.5 (528.7, 684.3)	495.9 (454.3, 537.4)	0.011
Mean change in 6MWT during pre-operative period (metres)	+ 85.6 (+ 18.1, + 153.2)	+ 13.2 (− 6.8, 33.2)	0.0135
Change in 6MWT during pre-operative period			
Improvement	9 (82%)	3 (27%)	
No change^a^	2 (18%)	7 (64%)	
Decline	0 (0%)	1 (9%)	

Data are presented as mean (95% CI) or *n* (%)

*6MWT* 6-min walk test, *HADS* hospital anxiety and depression scale

^a^“No change” represents participants whose pre-operative 6MWT distance was within ± 20 m of their baseline 6MWT distance

### Patient compliance

Prehabilitation group participants wore the Fitbit Charge 2 on 98.9% of days and control group participants wore the placebo Fitbit Flex 2 on 95.8% of days between enrolment and surgery. Compliance with the exercise component was high, with prehabilitation group participants achieving 30 min of moderate-intensity activity on average 59.9% (95% CI, 41.3–78.5) of the days during the pre-operative period, compared to 42.6% (95% CI 18.4–66.7) of days in the control group. As prehabilitation group participants were encouraged to engage in 30 min moderate-vigorous activity on at least 5 days a week, we can infer that compliance to the programme was 84% (i.e. 4.2 days). Comparatively if we apply the same criterion to the control group, “compliance” was 60% (i.e. 3.0 days). Compliance to resistance training component could not be objectively monitored via the Fitbit.

Usability of the Fitbit food log was also high, with prehabilitation group participants logging food on average 82.9% of the days. Participants on average only listened to the mindfulness App on 15% of days. Reasons for non-compliance to the mindfulness intervention included participants reporting good baseline mental health, finding the App unhelpful, and only using the App when they felt they needed to. Most participants (73%) already owned a suitable smartphone or tablet and the other 27% (six participants) were loaned a smartphone due to incompatibility of their own device with the FitBit, connection issues, or not owning a smartphone. One technical issue was reported during the study (a Fitbit Flex 2, which stopped charging). No adverse events were reported.

### Physical activity levels

Average daily minutes of vigorous intensity activity were significantly greater in the prehabilitation group [36.1 min (95% CI 21.2–50.9 min) vs 17.5 min (95% CI 5.2–29.7 min), *p* = 0.022]. Daily minutes of moderate-intensity activity were also higher in the prehabilitation group compared to the control group [25.1 min (95% CI 9.8–40.4 min) vs 13.1 min (95% CI 6.0–20.3 min), *p* = 0.063], although these results did not reach statistical significance. Daily minutes of light intensity activity were similar between the groups [208.3 min (95% CI 170.2–246.5 min) vs 205.3 min (95% CI 146.1–264.5 min), *p* = 0.925]. Mean daily step counts during the prehabilitation period were greater in the prehabilitation group compared to control group [8919 (95% CI 7024–10814) versus 7961 (95% CI 5314–10608), *p* = 0.519], although this was not statistically significant.

### Functional capacity

Table 2 presents the 6MWT distance at baseline and on the day before surgery for both study groups. The mean change in 6MWT distance for the prehabilitation group during the pre-operative period was + 85.6 m (95% CI 18.1–153.2 m) compared to + 13.2 m (95% CI − 6.8 to 33.2 m) in the control group (*p* = 0.014). All participants in the prehabilitation group improved their 6MWT distance, with nine participants (82%) increasing by 20 m or more. In the control group, eight participants increased their 6MWT distance, with only three (27%) participants improving by more than 20 m. In addition, three control group participants (27%) declined in 6MWT performance, with one participant (9%) declining by greater than 20 m.

### Body weight

Changes in body weight during the pre-operative period were minimal for both study groups. Mean change in weight in the prehabilitation group was + 0.46 kg (range − 1.0 to + 2.5 kg) versus − 1.06 kg (range − 3.85 to + 0.70 kg) in the control group. Qualitative analysis of the nutritional intervention identified that all prehabilitation participants used the food log on most days. Participants stated that the food log “gave me increased focus on my calorie control” and “helped to increase my food intake, but protein targets were difficult to stick to”, although two participants stated the app-based food diary was confusing and difficult to use.

### Psychological well-being

Mean HADS Anxiety Scale scores decreased slightly in both the prehabilitation and control groups. No significant difference in the reduction of HADS anxiety scores between the prehabilitation and control groups was found [− 0.5 (95% CI − 2.0 to + 0.9) versus − 1.2 (95% CI − 2.1 to − 0.2) respectively, *p* = 0.415]. Mean HADS Depression scores also decreased slightly in both the prehabilitation and control groups [− 1.4 (95% CI − 2.4 to − 0.3) vs − 0.8 (95% CI − 2.2 to + 0.5) respectively, p = 0.484]. Within-group analysis identified that the reduction in HADS Anxiety and HADS Depression scores were not significant in either group.

### Participant satisfaction

Following completion of the prehabilitation programme, participants completed an end-of-study questionnaire. Responses to this questionnaire are presented in Table 3. Overall, 100% of participants rated both the prehabilitation programme overall and the exercise component “Good” or “Excellent”. Ten participants (90%) responded “Strongly Agree” or “Agree” to the statement “The Fitbit motivated me to do the physical activity that was part of the prehabilitation programme”.

**Table 3:** Prehabilitation group participant’s responses to the End-of-Study Questionnaire

Item	Agreement rating					
	Excellent	Good	Average	Fair	Poor	Not answered
How would you rate the prehabilitation programme *overall*?	5 (45%)	6 (55%)	0 (0%)	0 (0%)	0 (0%)	0 (0%)
How would you rate the *exercise component* of the prehabilitation programme?	5 (45%)	6 (55%)	0 (0%)	0 (0%)	0 (0%)	0 (0%)
How would you rate the *dietary component* of the prehabilitation programme?	3 (27%)	5 (45%)	3 (27%)	0 (0%)	0 (0%)	0 (0%)
How would you rate the *mental well-being component* of the prehabilitation programme?	3 (27%)	3 (27%)	2 (18%)	0 (0%)	1 (9%)	2 (18%)

	Strongly agree	Agree	Neutral	Disagree	Strongly disagree	Not answered
The Fitbit Charge 2 was easy to use	9 (81%)	0 (0%)	1 (9%)	0 (0%)	0 (0%)	1 (9%)
The Fitbit Charge 2 was comfortable to wear	10 (90%)	1 (9%)	0 (0%)	0 (0%)	0 (0%)	0 (0%)
The Fitbit motivated me to do the physical activity that was part of the prehabilitation programme	5 (45%)	5 (45%)	1 (9%)	0 (0%)	0 (0%)	0 (0%)
The tailored exercise regime helped me to increase my exercise levels	5 (45%)	6 (55%)	0 (0%)	0 (0%)	0 (0%)	0 (0%)
Taking part in this programme has encouraged me to increase the amount of exercise I do once I recover from surgery	8 (72%)	2 (18%)	1 (9%)	0 (0%)	0 (0%)	0 (0%)

Data are presented as *n* (%)

In addition, participants in the control group were asked “The Fitbit motivated me to increase my physical activity although I was not asked to complete an exercise programme”, to which five (45%) participants stated they “Strongly Agree” and four (36%) participants stated they “Agree”, with two (18%) responding “Neutral”.

## Discussion

Prehabilitation aims to improve pre-operative functional capacity and “cardiopulmonary reserve” through pre-operative exercise, nutritional optimisation, and enhancing psychological readiness for surgery. The results of this study suggest that a programme of remotely supported tri-modal prehabilitation may increase pre-operative functional capacity before major abdominal cancer surgery. Smartwatches and associated technologies were used as the mainstay support for a prescribed programme of structured exercise/physical activity, optimal nutrition, and stress management. Participants allocated to prehabilitation increased their pre-operative 6MWT distance by an average of 85.6 m, with nine participants achieving an increase of > 20 m, suggesting a clinically important improvement in functional capacity. A previous RCT has demonstrated marginally greater increases in 6MWT distance in an exercise prehabilitation group compared to a control group (+ 31 m vs + 27 m) following home-based prehabilitation; however, this study required a physical therapist to make regular home visits (on six occasions over 4 weeks) to ensure compliance to the exercise programme [29]. Conversely, Carli and colleagues found a reduction in 6MWT distance in a bike/strengthening group (− 6.8 m) following home-based prehabilitation and compliance rate of only 16% despite home visits and weekly phone calls [5]. Our study has demonstrated that functional walking capacity may be increased to a desired threshold with prehabilitation delivered through a wearable technology and smartphone application. The acceptability and compliance with this device and its associated exercise intervention was high with patients reaching 30 min moderate-intensity activity on 84% of days they were encouraged to do so. This compares to compliance rates of 16–87% in previously reported pilot studies of home-based prehabilitation [4].

Daily activity levels were higher in the prehabilitation compared to the control group, with the latter completing significantly more minutes of vigorous daily exercise and increased minutes of moderate daily exercise. Feedback from end-of-study questionnaire suggests that the Fitbit wearable devices were an independent factor responsible for encouraging participants to complete the prescribed exercise by providing easily visible feedback on progress towards activity goals.

The nutritional intervention aimed to prevent unintentional weight loss prior to surgery which is particularly important in cancer patients. The relatively high baseline BMI of our groups (27–30 kg/m^2^) reflects the nutritional and body mass status of patients in our UK population, although it should be noted that BMI is itself a crude measure of nutrition. One of the limitations of prehabilitation before cancer surgery is there are not more than 2–3 weeks in which to deliver a nutritional intervention. Changes in body weight from baseline to the day before surgery were minimal in both groups but this intervention may be more relevant for frailer patients to prevent further weight loss before surgery and warrants further review. Qualitative comments suggest that the protein targets were challenging for patients, suggesting the use of a food log App may need further development to improve efficacy in this setting. Oral Nutritional Supplementation has previously been shown to reduce post-operative length of stay following colorectal surgery [19] and could be used alongside dietary advice in frailer patients to enhance the dietary component of our prehabilitation programme. The incorporation of a nutritional status questionnaire, such as the Nutrition Risk Screening Tool 2002 [30], into baseline assessments could identify those patients at greatest risk for weight loss and malnutrition and ONS could be provided to these at-risk patients.

The psychological intervention aimed to reduce pre-operative stress and anxiety by teaching mindfulness stress management techniques. In-group analysis revealed that reductions in anxiety and depression in the prehabilitation group were not statistically significant. This may reflect poor compliance with the psychological intervention or poor efficacy of the mindfulness app in this context, suggesting further development of the mindfulness component may also be needed.

Several limitations of this pilot study should be noted. Firstly, participants could not be completely blinded to their allocated study group. This may have generated bias as participants in the control group were aware that we were investigating the impact of pre-operative exercise and may have altered their exercise levels accordingly. The control group were provided with a placebo Fitbit Flex 2 (no display screen or feedback), with our end-of-study questionnaire suggesting 82% of control group participants stated that they increased physical activity just because they were given a placebo device to wear. This demonstrates how even just wearing a smartwatch can increase motivation for physical activity. It is important to note that we did not exclude patients who were already completing the national recommendation of 150 min physical activity per week, but even these patients reported enjoying participation and that it “gave them something to focus on”, with a beneficial effect on their mental health. Finally the sample size in this study was small with a risk of Type 2 error.

To our knowledge, this is the first pilot study to demonstrate the efficacy of delivering home-based prehabilitation prior to major abdominal cancer surgery using wearable smart technologies, with clinically and statistically significant improvements in functional walking capacity and higher levels of vigorous physical activity in the prehabilitation group when compared to controls. This study has demonstrated that the delivery of home-based prehabilitation using smartwatches and mobile applications is well-accepted in patients awaiting major abdominal cancer surgery, and that the primary outcomes of 6-min walk test (6MWT) distance and levels of activity as measured by wearable devices may be reliably used in future trials. We feel that further optimisation of the nutritional and psychological components is required, perhaps through further integration into a single smartphone application. A fully powered RCT is warranted to investigate the effects of a prehabilitation programme on post-operative outcome measures such as length of hospital stay and complication rates in this setting.

## Supplementary Information

Below is the link to the electronic supplementary material.

Supplementary file 1 (DOCX 17 KB)
