# Supplementary material for: Prehabilitation with wearables versus standard of care before major abdominal cancer surgery: a randomised controlled pilot study (trial registration: NCT04047524)
Source: Surg Endosc. 2021 Mar 15;36(2):1008–17. doi: 10.1007/s00464-021-08365-6 (PMC8758615; doi:10.1007/s00464-021-08365-6)
Supplement: Supplementary file 1 — (DOCX 17 KB) [file 464_2021_8365_MOESM1_ESM.docx]

Appendix 1:

**Nutrition Information Sheet**

A Tri-modal Prehabilitation Programme using Smart Watches and Mobile applications in Patients undergoing Major Abdominal Surgery: a randomised controlled pilot study.

**Eating a balanced diet**

If you are eating well and have not lost any weight recently it is important to maintain a stable weight to aid recovery and allow your body to heal after your operation.

Try to eat a balanced and nutritious diet leading up to your surgery. You can do this by eating 3 meals a day, and snacks if required, which should include:

1. **A protein source** at each meal, for example chicken, turkey, meat and meat products*, fish, eggs, milk and dairy, beans**, lentils**, pulses**, soya and soya products, tofu and nuts**.

*Red meats (for example beef, lamb, pork and goat) are an excellent source of iron and can be beneficial to include in your diet, however the recommendations are to limit the amount consumed.

It is also recommended to avoid processed meats (preserved by smoking, curing, salting or the addition of chemical preservatives, such as ham, salami, bacon and frankfurter sausages). Processed meats will also be lower in lean protein too!

Protein is essential for growth and repair of your body.

1. **A starchy food source** with each of your meals for example bread, chapatti, cereals, rice, pasta, crackers, oatcakes, quinoa**, cous-cous, noodles, potatoes**. Aim to choose wholegrain varieties where possible**.

Carbohydrate is the main energy source for body to promote healing after surgery.

1. **Aim to a variety eat fruit and vegetables**** including green, purple, red, orange, yellow and white (this includes fresh, tinned, dried or juiced).

Fruit and Vegetables are a good source of vitamins, minerals and phytochemicals which can help your body to defend itself and help support your immune system.

**If you have experienced diarrhoea and/or severe wind and bloating just eat higher fibre foods as tolerated as you may find limiting higher fibre/wholegrain foods helps with your symptoms.

**If you have been advised to follow a low fibre diet by your doctor or dietitian then refer to The Christie booklet ‘Eating well when following a low fibre diet’ for advice on what to eat (Please ask and you will be given this booklet).

**Alcohol**

- Government guidelines suggest for both men and women it is safest not to drink more than 14 units of alcohol a week on a regular basis. If you regularly drink as much as 14 units per week, it's best to spread your drinking evenly over three or more days. For more information please visit the <https://www.drinkaware.co.uk/> website.

**Weight loss and poor appetite**

If you are concerned that you are losing weight or are suffering with a poor appetite, then you may need to increase energy and protein in your diet. This may involve changing the balance of what you eat by choosing foods that are higher in fat and sugar than the government or World Research Cancer Fund would normally advise.

If you lose weight, especially over a short period of time, it is associated with muscle loss. This may cause you to have low energy levels, reduced strength and an impaired ability to perform daily tasks. By eating a higher energy and protein diet and doing physical activity, as advised by the physiotherapist, you can help to minimise muscle loss and aim to build muscle strength you may have already lost.

By eating as well as you can, you are giving your body the nutrients it needs to rebuild damaged tissue, fight infection and cope with any side effects of your surgery.

For more information you can read our guide for patients with eating problems – ‘Eating – Help Yourself’. <http://www.christie.nhs.uk/media/2400/218.pdf>. This can also be found at the Patient Information Centre.
